# Supplementary material for: Are carbon emissions trading and green financial instruments synergistic? -Comprehensive quantitative research based on content analysis
Source: PLoS One. 2024 Mar 7;19(3):e0298601. doi: 10.1371/journal.pone.0298601 (PMC10919601; doi:10.1371/journal.pone.0298601)
Supplement: S1 Data — (ZIP) [file pone.0298601.s001.zip › Data/地级市Z_F工作报告.zip!/╜¡╦╒/╨∞╓▌╩╨/└┤╫╘╣1⁄2╓┌║┼í╛┬φ┐╦╩2╛▌═°í┐_2018.docx]

各位代表：

现在，我代表市人民政府向大会作工作报告，请予审议，并请市政协各位委员和其他列席人员提出意见。

一、2017年工作回顾

2017年，是徐州发展进程中极不平凡的一年，也是各项工作取得重大突破的一年。一是较好完成了十六届人大一次会议确定的目标任务。在省委省政府和市委的坚强领导下，全市上下认真贯彻落实习近平新时代中国特色社会主义思想，聚焦打造淮海经济区中心城市，凝心聚力、开拓奋进，切实推进各项重点工作落实，全年预计（下同）实现地区生产总值6600亿元、增长8%左右，一般公共预算收入完成501.6亿元、同口径增长5%，其他各项主要指标增速高于全省平均水平，经济社会发展保持了稳中有进、稳中向好的态势。二是办成了一些过去想办而未能办成的大事。经过全市上下的共同努力，年初我市荣获中国人居环境奖，11月跻身全国文明城市行列，12月成为“中国最具幸福感城市”之一，为城市发展再添一批高含金量的靓丽名片。6月国务院批复徐州城市总体规划，明确了我市淮海经济区中心城市的定位；7月省委常委会专题研究徐州工作，把徐州建设淮海经济区中心城市纳入全省“1+3”重点功能区战略，8月省委省政府专门出台政策意见予以支持，徐州的发展定位被提到了前所未有的战略高度。三是取得了一批开创性的丰硕成果。首次成功举办国际服务外包大会、国际物流大会等国际性会议；全国“城市双修”工作现场会、城市管理工作现场会、商务综合行政执法体制改革现场会等重要会议先后在我市召开；徐州综合保税区通过审批，国际邮件互换局兼交换站开工建设，国际互联网数据专用通道开通，这些工作对我市当前和长远发展都将产生深远而积极的影响。

特别是12月12日、13日，习近平总书记亲临徐州开展十九大后首次调研视察，对我市振兴转型发展实践给予充分肯定，为徐州决胜全面建成小康社会、开启现代化新征程指引了前进方向、注入了强大动力，这是我市发展史上具有里程碑意义的一件大事，全市上下群情振奋、无比喜悦，受到巨大的鼓舞和激励。

徐州一年来的变化是全方位、深层次、开创性、历史性的变化，城市的知名度、美誉度、影响力显著提升，群众的获得感、幸福感、自豪感明显增强！

一年来，我们主要做了以下工作：

（一）坚定不移走产业强市之路。强化产业发展导向，深入实施“工业立市、产业强市”战略，以产业振兴带动全面发展。产业转型升级步伐加快。工业经济在狠抓传统产业改造提升的基础上，加快培育新兴主导产业，出台装备与智能制造、新能源、集成电路与ICT、生物医药四大新兴主导产业实施方案，制定特色产业基地发展实施意见，建立部省安全产业共建培育机制。规上工业增加值增长9%左右，增幅居全省前列；新兴产业产值占比达到33%，较上年提升3.6个百分点；高新技术产业产值增长15%左右，占比达到37%。服务业重点发展现代物流、服务外包、金融服务等业态，服务业增加值增速高于GDP增速0.5个百分点、占比提升至47.3%，实现社会消费品零售总额2970亿元、增长12%。粮食生产实现“十四连丰”，高效设施农业规模、占比继续保持全省第一，建成上海蔬菜外延基地9家；我市获批全国首批农业可持续发展试验示范区。黄河故道综合开发取得显著成效，当年实施项目82个，完成投资48亿元。重大产业项目进展良好。全年实施产业项目4656个，比上年增加1359个。鑫华半导体级多晶硅、东方金钰智能机器人等项目建成投产，台湾正崴高端手机配套链、茂迪太阳能电池、深圳正威新材料、协鑫大晶圆及配套、招商局集团投资建设徐州金融集聚区、中国电子集团信息产业基地、林安物流园等一批投资百亿级项目签约落地。固定资产投资完成5275亿元，增长10%，其中二三产业投资增长10.1%。经济运行质量稳步提高。规上工业企业利润总额增长18%，工业、服务业应税销售收入分别增长24.7%和35.8%。工商新登记企业4.31万家，增长16.1%。金融机构新增存款近1000亿元，新增贷款超过500亿元，增速均居全省前列。

（二）全力打造淮海经济区中心城市。推动“靓丽徐州”向“品质徐州”提升，不断增强集聚辐射带动能力。中心城市定位更加明晰。围绕国家历史文化名城、全国重要综合性交通枢纽、淮海经济区中心城市和“一带一路”重要节点城市“四大定位”，制定实施经济、商贸物流、金融服务、科教文化“四个中心”建设三年行动纲要，为城市长远发展明确坐标。成功争取中心城区土地利用规划范围调整，编制完成清风廊道、综合管廊等详细规划，城市发展空间进一步拓展。城市功能日趋完善。围绕中心城市交通枢纽、快速交通体系、中心商圈、教育文化卫生功能提升，实施了一批城市重大基础设施和功能性项目。150项城建重点工程开工134项、竣工52项。徐连客专、徐宿淮盐铁路、观音机场二期建设有序推进，徐明高速公路（江苏段）建成通车。轨道交通1、2、3号线一期工程稳步实施，迎宾大道高架、城东大道高架、外环公路等道路开工建设，319条市内道路完成整修，城区快速路网体系加快形成。苏宁广场、三胞广场相继营业，建成国家物流标准化试点城市，中心商圈辐射力进一步增强。信息基础设施日益完善，城域网出口带宽达3660G。出台中心城区中小学校布点专项规划，一中新校区完成主体建设，市第一人民医院整体南迁、北区股份制医院建设进展顺利，优质公共资源分布更加均衡、服务水平不断提升。实施棚户区改造1610万㎡。505个老旧小区实施综合整治，20万户62.5万市民居住环境焕然一新。城市品质加快提升。以创建全国文明城市为主抓手，努力提升城市品质和文明程度，市容环境治理、交通秩序整治、文明志愿服务、道德模范和身边好人评选深入开展，文明风尚蔚然成风，市民文明素质明显提升，19家单位获得全国文明单位称号，群众对文明创建的认可度和满意率明显提高。顺利通过国家卫生城市复检，获批全国第二批城市设计试点城市、第三批生态修复城市修补试点城市。城市绿色生态建设又有新进展，梨园路公园、五山公园一期建成开放，泉润公园、拖龙山山体公园等精品园林建设有序推进。城市长效化管理机制不断完善，占道经营、渣土运输、违法建设等专项整治成效明显，徐州“百姓城管”经验在全国推广。年内来徐游客达到6903万人次，增长45.5%。新型城镇化试点有序推进。以新型城镇化推动城市功能拓展、带动城乡统筹发展，9个新型中心镇（小城市）、15个新型社区完成规划编制，特色小镇、“百村示范”、“千村提升”和特色田园乡村试点进展顺利，31个美丽康居村庄和460个美丽宜居村庄基本实现创建目标，14个村镇获全国文明村镇称号。

（三）积极培育发展新动能。高度聚焦改革开放创新，将加快动能转换推动发展放到重中之重的位置，取得重大进展。各项改革全面深化。突出“三去一降一补”政策落实，累计关闭矿井13对、压减煤炭产能1062万吨、压减钢铁产能163万吨，商品住房去化周期由15.32个月下降到7.36个月，全年降低实体经济企业成本92.6亿元，补短板专项工程完成投资510亿元。扎实推进“放管服”改革，在全省率先出台“3550”标准化流程，“不见面”审批清单实现全覆盖，相对集中行政许可权改革试点稳步实施。统筹抓好其他改革，河长制全面推行，服务业综合改革、不动产登记、水流产权确权等一批特色改革试点工作走在全国全省前列。创新驱动战略深入实施。“一城一谷一区一院”等科技创新平台建设加快推进，江苏淮海科技城入驻企业达到3000多家，徐州科技谷进驻院士团队2个、企业110余家，潘安湖科教创新区开工建设，产业技术研究院集聚各类研究机构近20家。科技创新体系逐步完善，鼓楼高新区获批筹建，云龙电商科技园等一批创新园区相继开园；新增国家高新技术企业140家、国家级科技企业孵化器2家、省级以上研发机构33家；完成专利授权量1.1万件，其中发明专利授权量1600件。“彭城英才”计划深入推进，“赢在徐州”大赛影响力持续提升，入选国家和省人才项目创历史新高。金融创新力度加大，新引进各类金融机构98家，华信新材料在深交所上市，新增“新三板”上市企业4家，新增江苏股交中心挂牌企业420家，新增企业债66.9亿元、股权融资35亿元，落地产业基金31支、首期母基金规模518亿元，平安银行、恒丰银行和苏北首家离岸金融业务中心开业运营，金融集聚区启动规划建设，多层次资本市场框架初步构建。徐工集团获批国家级制造业与互联网融合、工业互联网应用试点示范，中能硅业喜获“省长质量奖”。我市获批创建全国质量强市示范城市。双向开放实现新突破。开放平台建设成效显著，电子口岸上线运行，跨境电商线上综合服务平台试运行，“无水港”启动运行，“外贸港”建设快速推进，中欧班列开行50班。成功举办“二十洽”、彭城英才荟等17项重大招商活动，新引进总投资5亿元以上内资项目140个、3000万美元以上外资项目33个，项目投资总额、质量显著提高。实际使用外资完成16.6亿美元、增长10.2%，进出口总额、出口总额分别达到537亿元和439亿元，增长30.1%和26.1%，服务外包合同额、执行额分别增长2倍和1.5倍，增速均居全省前列。

（四）持续推进生态文明建设。以高度的自觉严守生态红线、实施治理修复、提升环境质量，认真抓好中央和省环保督察反馈问题整改，探索生态优先、绿色发展的新路子。积极参与江淮生态大走廊建设。在全省率先开展专题研究、编制专题规划、制定实施方案，构建“两轴、两湖、多片区、多点”生态空间结构，着力打造“五个走廊”。认真落实“263”专项行动。累计关闭化工企业143家，减少煤炭消费量679万吨。39条黑臭河道治理基本完成，Ⅲ类以上水体占比达到79.2%，国考断面水质100%达标。全市划定畜禽禁养区。275家挥发性有机物重点企业治理基本完成。关停整治小砖瓦企业144家。狠抓生态治理和节能减排。扎实推进生态修复，潘安湖湿地被列入全国首批国家湿地旅游示范基地，九里湖湿地获批国家湿地公园。强化大气污染综合管控，完成2798台燃煤小锅炉拆除改燃，拆解老旧运输船舶380艘，淘汰黄标车5742台，秸秆禁烧成效明显。大力调整能源结构，单位GDP能耗下降7%左右，超额完成省定任务。实施519项重点治污工程，污染物排放总量削减完成年度任务。丁万河获评全国首届“最美家乡河”。我市成为全国首批、江苏首家水生态文明城市。

（五）尽心尽力改善民生民计。坚持民生为本，以扎实举措不断增加民生福祉。民生实事项目基本完成。十大类70件民生实事有力推进。为82.47万老人、2.55万残疾人（儿童）办理意外伤害保险，发放困难残疾人生活补贴和重度残疾人护理补贴2.4亿元。城乡供水一体化建设累计完成投资65.7亿元。建成农村公路1082公里、桥梁407座，完成改厕39925户。新建改造15家街坊中心及农贸市场，建成100个标准化社区居家养老服务中心，完成52个镇（街道）综合文化站、518个村（社区）综合文化服务中心达标建设。富民增收工程深入实施。城乡居民收入分别增长9%和9.3%。城镇新增就业12万人、失业人员再就业10.1万人，城镇登记失业率控制在1.82%。19.5万低收入人口实现脱贫，68个经济薄弱村集体经济收入达到18万元以上，123个经济薄弱村综合服务中心建成使用，湖西片区帮扶初见成效。社会保障水平逐步提高。城乡居民基本医疗保险制度整合实施，基本照护保险、机关事业单位养老保险制度改革有序推行，在全省率先实现跨省异地就医结算。退休人员人均基本养老金、最低工资、城乡低保、农村五保等标准继续上调，在全省率先将60多万建档立卡低收入家庭纳入医疗救助范围。各项社会事业全面发展。新改扩建中小学119所、幼儿园184所，创建义务教育标准化学校185所、省现代化示范性职业学校3所。“健康徐州”建设加快推进，省级综合医改试点、分级诊疗制度有序实施。现代公共文化服务体系、养老服务体系不断完善。圆满举办首届徐州国际马拉松赛和国际龙舟邀请赛，徐州籍体育健儿在全运会取得历史最佳成绩。社会治理能力不断增强。“四项督查”常态开展，“百千万”大走访活动深入实施，累计走访群众209.4万户、企业4.4万家，解决问题10.6万个。平安法治徐州建设扎实推进，矛盾纠纷多元化解机制不断健全，社会治安防控、应急管理体系进一步完善，开展三轮打黑除恶围猎专项行动，信访总量、刑事案件立案数、安全生产事故分别下降31.8%、16.4%、25.1%，社会大局保持和谐安定。人防、国防动员、双拥、民族宗教、档案、外事、侨务、台湾事务、审计、统计、物价、新闻出版、机关事务管理、住房公积金管理、无线电管理、淮塔管理、防震减灾、气象、老龄、青少年、妇女儿童、红十字、关心下一代等各项事业都取得了新进步。

（六）构建高效廉洁的服务型政府。坚持以更高标准加强政府自身建设，不断提高政府的公信力执行力凝聚力。政府组织工作水平持续提升。长效执行市政府周工作例会、重点工作定期调度、督查督办等制度。深入推行县（市）区重点经济工作、部门创新奖、干部作风绩效、社会治理重点工作“四大考核”。出台支持徐州经济技术开发区、高新区创新发展政策。调整完善市区两级财政管理体制、城市建设和管养体制、土地储备和市场规范运作机制，成立PPP和产业基金办公室，实现了政府组织推进经济社会发展的体制机制、方式方法的转换。举办第二届“徐州经济年度人物”和“十佳优秀企业家”评选，弘扬了企业家精神，提振了企业发展信心。政务服务水平明显提高。坚持依法履职，加快打造法治政府。“334”改革大力推进，新政务服务中心投入使用，审批部门、行政许可事项实现集中进驻，审批时限压减幅度达到57%，政务服务网服务覆盖面、“12345”政府服务热线建设全省领先，公共资源交易平台实现一体化管理。政风建设不断加强。主动接受人大工作监督和法律监督、政协民主监督、社会监督，办理市人大代表议案建议381件、市政协提案531件，办结率100%，满意率99%。切实抓好“两学一做”学习教育常态化制度化，深入开展“三清示范区”建设，严格落实中央八项规定精神和省、市委十项规定，强化党风廉政建设责任制，落实“一岗双责”，加大“四风”问题监督检查力度，加强行政监察和审计监督，严管公共资金、公共资源和国有资产资本，保持反腐败斗争高压态势，政务环境得到进一步优化。

各位代表！过去一年的成绩来之不易、影响长远，是市委坚强领导的结果，是全市上下和社会各界团结奋斗的结果。在此，我代表市人民政府，向全市人民，向各位人大代表、政协委员，向各民主党派、工商联、无党派人士，向各人民团体、各界人士，向驻徐人民解放军、武警官兵和人民警察，向关心和支持徐州建设的香港特别行政区同胞、澳门特别行政区同胞、台湾同胞、海外侨胞和国际友人，表示衷心的感谢和崇高的敬意！

我们也清醒地看到，我市经济社会发展中还存在不少问题和困难：发展不充分不平衡的问题比较突出，发展质量和效益还不高；产业层次有待进一步提升，转型升级任务依然艰巨；中心城市辐射带动力需要进一步增强，城乡二元结构突出，农村面貌有待加快改善；民生领域存在不少短板，公共服务与群众期待相比还有差距，社会治理基层基础工作仍需加强；生态欠账依然较多，大气、水、土壤等环境质量亟待改善；一些政府工作人员服务和担当意识不强，等等。我们一定高度重视这些问题，采取更加有力措施，切实加以解决。

二、2018年工作指导思想和主要预期目标

2018年是贯彻党的十九大精神的开局之年，是改革开放40周年，也是决胜全面建成小康社会、实施“十三五”规划承上启下的关键一年。在深入学习贯彻十九大精神的重要时刻，习近平总书记亲临徐州调研视察，对发展实体经济、抓好创新驱动、推动乡村振兴、建设生态文明、加强基层党建等作出重要指示、提出明确要求。这是习近平新时代中国特色社会主义思想在徐州的具体化，也是我们在新时代决胜全面建成小康社会、开启现代化建设新征程的根本遵循。我们要把学习贯彻党的十九大精神作为首要政治任务，把贯彻落实习总书记视察徐州重要指示作为推进新时代徐州发展的最大历史机遇，坚决维护以习近平同志为核心的党中央权威和集中统一领导，始终在思想上政治上行动上同党中央保持高度一致，紧密结合徐州实际，不忘初心、牢记使命，抢抓机遇、埋头苦干，不断把徐州各项事业推向前进，确保党的十九大精神和习总书记视察徐州重要指示全面落地生根、开花结果。

今年政府工作的指导思想是：深入学习贯彻党的十九大精神和习近平总书记视察徐州重要指示，以习近平新时代中国特色社会主义思想为指导，坚持稳中求进工作总基调，坚持新发展理念，按照高质量发展的要求，统筹推进“五位一体”总体布局和协调推进“四个全面”战略布局，以供给侧结构性改革为主线，以建设淮海经济区中心城市为重点，统筹推进稳增长、促改革、调结构、惠民生、优生态、防风险各项工作，促进经济平稳健康发展和社会和谐稳定，加快建设“强富美高”新徐州。

今年经济社会发展的主要预期目标是：地区生产总值增长8%左右，一般公共预算收入增长8%左右，固定资产投资增长10%以上，社会消费品零售总额增长12%左右，进出口总额和实际使用外资分别增长10%以上，科技研发投入占比达到2.3%左右，居民收入增长高于GDP增长，居民消费价格涨幅控制在3%左右，城镇登记失业率控制在4%以内，节能减排完成省下达任务。

贯彻指导思想，实现预期目标，我们将坚定不移做到“五个坚持”。一是坚持认真贯彻新时代战略安排。深刻把握新时代的历史内涵和实践要求，按照新时代徐州发展的总定位总要求和“三步走”的战略步骤，紧盯“十三五”后三年发展目标，坚决打赢全面建成小康社会决胜仗，探索开启建设现代化新征程。二是坚持以人民为中心的发展思想。准确把握社会主要矛盾变化，把人民对美好生活的向往作为奋斗目标，更加突出抓重点、补短板、强弱项，一件事情接着一件事情办，一年接着一年干，着力解决发展不充分不平衡问题，更好推动人的全面发展、社会全面进步。三是坚持大力推动高质量发展。按照推进高质量发展这一根本要求，进一步匡正发展理念、转变发展方式，坚持稳中求进工作总基调，坚持质量第一、效益优先，大力推动质量变革、效率变革、动力变革，系统谋划打好“三大攻坚战”等重点工作，努力在高质量发展上不断取得新进展。四是坚持深入实施创新驱动战略。把创新作为引领发展的第一动力，大力推进科技创新、机制创新、方法创新，加快集聚人才、科研及科技成果等创新要素，努力建设区域领先的创新高地。五是坚持高度聚焦中心城市建设。以现代化理念谋划淮海经济区中心城市建设，集中精力打造经济、商贸物流、金融服务、科教文化中心，全面提高城市首位度，努力建设让徐州人幸福自豪、让外地人羡慕向往的中心城市。

三、2018年政府工作的主要任务

新的一年里，我们要以党的十九大精神和习近平总书记视察徐州重要指示为指引，科学施策、务实苦干，持续提升徐州的产业支撑力、城市承载力、发展竞争力和辐射带动力。重点抓好以下六个方面的工作：

（一）深化供给侧结构性改革，大力推进经济高质量发展。习总书记在徐州视察时特别强调，要始终高度重视发展壮大实体经济，经济要由高速增长向高质量发展转变。凸显实体经济，加快转型升级，不断优化产业结构，推动产业产品加快迈向中高端，建设现代化经济体系，将是当前和今后一个时期实现经济高质量发展的重要任务。

继续推进供给侧结构性改革。坚持用市场化、法治化手段推进“三去一降一补”。严格执行环保、质量、安全等相关法规和标准，大力化解过剩产能、淘汰落后产能；巩固清理“地条钢”成果，防止死灰复燃；加大“僵尸企业”处置力度，妥善安置好企业职工。科学把握土地、金融、财税等调控政策措施，加快建立长效机制，促进房地产市场供需平衡、健康发展。稳步推进多层次资本市场建设，进一步提高直接融资比重，积极稳妥降低企业杠杆率。认真落实降低企业成本各项政策，在降低制度性交易成本、税负成本和要素成本上协同发力，进一步降低企业用能、物流、融资等成本；继续清理规范涉企收费，加大对乱收费的查处和整治力度。围绕创新、富民、基础设施等重点领域，继续实施一批补短板项目。

持续做大做强主导产业。以提高质量效益、增强核心竞争力为目标，支持传统产业抓好技术升级、设备更新、“两化融合”和绿色低碳改造，推动互联网、大数据、云计算、人工智能和实体经济融合，促进“四新经济”加快发展，完善“6+6”先进制造业体系和“333”现代服务业体系。深入落实装备与智能制造、新能源、集成电路与ICT、生物医药产业实施方案，抓好自主创新和关键技术攻关，尽快培育四大千亿级主导产业集群。突出发展供应链物流、保税物流等特色物流和批发零售、电子商务、服务贸易等商贸业态，加快专业市场搬迁改造升级，构建大市场大物流，打造区域性商贸物流中心。加快构建多元化现代金融体系，抓好新城区金融集聚区、开发区基金小镇建设，大力发展金融服务业，发挥好产业基金引导作用，打造服务淮海经济区的金融高地。推进“旅游+”融合发展，争创国家全域旅游示范市。开展质量提升行动，弘扬劳模精神和工匠精神，创建全国质量强市示范城市，打响“徐州制造”品牌。积极争创中国制造2025国家级示范区、国家老工业城市和资源型城市产业转型升级示范区。

集中精力主攻重大项目建设。坚持把项目建设作为发展的“牛鼻子”、产业振兴的主抓手，全力推进总投资4662亿元的210项重大产业项目建设。落实月调度、季观摩、半年督查、全年考核的项目推进机制，重点抓好淮海控股新能源汽车、茂迪太阳能电池、台湾正崴高端手机配套链、深圳正威新材料、复星医药、必康医药综合体等先进制造业项目，盒子支付、溢美金融、金鹰二期、全息科技文旅城、林安物流园、蓬莱极地海洋馆等现代服务业项目，中科曙光智能装备集成产业园、华信临港产业园、华融中财产业园、装配式建筑产业园等平台载体项目，抓好中建材薄膜电池等前期推进项目。通过强化招商引资、企业兼并重组等措施，力争再引进一批龙头型、基地型、旗舰型项目。

营造产业发展良好环境。在产业平台建设上下功夫，提升徐州经济技术开发区、高新区创新发展水平，支持有条件的园区创建国家级、省级开发园区，形成由开发区、高新区、南北共建园区、跨境跨区合作园区、特色园区构成的开发园区体系；加快建设潘塘物流园、北三环物流集聚带、物流大数据中心等物流服务平台，大数据产业园、智慧信息产业园等信息平台，国家智能工程机械质检中心和省特种机器人质检中心等检验检测平台。在政策支持上下功夫，加大对实体经济服务支持力度，强化资金、土地、能源等要素保障；全面实施市场准入负面清单制度，清除妨碍统一市场和公平竞争的各种壁垒；健全完善、及时兑现招商引资、支持现代产业发展和人才引进等政策措施。在优化服务上下功夫，健全制度化的政企互动机制，构建亲清新型政商关系，及时帮助企业解决实际困难，打造一流营商环境；实施名企名品名企业家工程，弘扬优秀企业家精神，营造尊崇企业家氛围，支持民营企业发展，着力培育一批行业领军企业、标杆企业和龙头企业。

（二）加快建设淮海经济区中心城市，提升城市承载力集聚力。中心城市不是领导“封”出来的、更不是自己喊出来的，是要靠实实在在干出来的。只有经济实力强了、城市能级品质高了、首位度提升了，我们才会有底气，别人才能够服气，这才是淮海经济区真正的“CBD”。

全面提升城市功能。注重传承文脉、彰显内涵，深度挖掘汉文化、彭祖文化、红色文化、书画文化等特色文化资源，展现“楚韵汉风”的独特魅力，建设有地域特色、有文化特质、有时代特征的现代化城市。统筹文、美、产、居等多种要素功能，开展徐州2049空间战略规划研究，启动新一轮城市总体规划修编，大力推进“多规合一”。围绕巩固综合交通枢纽地位，加快建设“米字型”高铁枢纽，实施铁路物流（专用线）发展规划，做好徐连运河规划对接申报，积极推进徐州东部绕越高速公路、睢宁至台儿庄高速公路规划建设，构建大交通格局。逐步打通河海统筹、亚欧陆桥、空中走廊等物流通道，构建国家级综合物流枢纽和多式联运中心、“一带一路”核心物流节点城市。实施科教文化提升工程，启动规划建设文化艺术中心、大剧院、科技馆、会展中心、公共卫生服务中心等一批一流的功能性项目，增强城市优质公共服务供给能力。持续抓好城市“双修”，积极申办中国国际园林博览会，确保通过国家森林城市新一轮复检考核，加快联合国人居环境奖创建步伐，彰显山水园林城市新形象。

完善城市现代基础设施。以形成更加配套、更加完善的基础设施体系，增强城市综合承载能力，强化同周边城市互联互通为重点，实施总投资2820亿元的六大类170项城建重点工程。加速构建城市快速交通体系，加快轨道交通建设进度，抓好徐贾快速通道南延、徐沛快速通道铜山段等区域间路网建设，继续实施市内道路综合改造，切实提高市民出行便利性。完善信息网络基础设施，重点推进光网徐州、无线徐州、“三网融合”、信息安全工程，积极打造“智慧徐州”。抓好海绵城市、综合管廊建设，统筹实施智能化电网、街坊中心建设、商品交易市场升级等工程。实施城镇防洪排水工程、郑集河输水扩大工程，加大市区易淹易涝片区治理。年内基本完成城乡一体化供水，让城乡居民一起喝上安全放心水！

不断提高城市治理水平。巩固全国文明城市创建成果，落实常态长效机制，提高城市文明程度，让文明城市这块金字招牌更有含金量。打响为百姓服务、请百姓参与、让百姓满意的“百姓城管”品牌，深化城管综合执法体制改革，扎实抓好城市出入口环境综合整治、建筑外立面治理，实施公厕新建改造、城乡生活垃圾分类处置、照明亮化提升、背街小巷整治等工程，打造精致、细腻、整洁、有序的城市环境。完成市区老旧小区三年整治任务，实施新一轮老旧小区改造提升工程，积极推进“煤改电”采暖和增设外挂电梯，实施2127万㎡棚户区及城中村改造，确保市民生活环境不断改善。抓好城市中心以及公园、学校周边停车场建设，年内增设5000个公共停车泊位，打造智慧化停车管理平台，逐步解决停车难问题。

（三）大力实施乡村振兴战略，构建城乡一体、统筹发展的新格局。按照习总书记提出的“产业兴旺、生态宜居、乡风文明、治理有效、生活富裕”的总要求，积极推进乡村振兴战略。加快农村新型城镇化，持续增加农民收入，逐步破解城乡二元结构，促进城乡融合发展。

加速推进农业现代化。大力推进农村一二三产业融合发展，加快构建现代农业“483”产业体系，抓好国家农业可持续发展试验示范区建设，促进传统农业向现代农业转变。积极稳妥推进农村土地承包经营权流转，发展农业适度规模经营。抓好上海蔬菜外延基地建设，强化现代农业示范园、特色农产品加工集中区建设和产业集群培育，提升农产品市场竞争力。筹建淮海经济区农业科技创新与转化联盟，健全科技为农服务体系。实施黄河故道综合开发和丘陵山区整体开发，加强耕地保护，抓好高标准农田建设，推进粮食生产全程机械化，探索开展良田专业化托管等社会化服务，进一步提高土地产出效益。

加快新型城镇化进程。坚持以人的城镇化为核心，完善“1530+30+130”新型城镇体系，提高城镇化发展质量。加快县级中等城市建设，提高综合承载功能、公共服务能力、现代管理水平，促进县城规模适度扩张。扎实推进重点中心镇建设，尽快建成一批领先苏北的小城市样板。依托产业特点、人文底蕴和生态禀赋，坚持差异化发展、特色化培育，打造一批国家级、省级特色小镇。

着力建设美丽宜居乡村。突出新型社区和特色田园乡村创建，因地制宜建设一批体现田园风光、乡村风情、乡土韵味的特色乡村。创新农村公共服务设施投入、建设、管护机制，加强农村医疗卫生、文化体育设施建设，促进优质公共服务向农村延伸。实施农村公路提档升级三年行动计划，抓好撤渡建桥，年内新改建道路500公里、桥梁100座，实现村级道路建设新突破。实施村庄环境改善提升行动，完善农村环卫设施，健全城乡垃圾收运处置体系，抓好农村改厕，强化农村水环境、生活污水整治，加大村庄绿化和河塘清淤力度，塑造洁净有序、水清树绿的村容村貌。加强农村（社区）治理，抓好农村文化建设和精神文明建设，培育邻里和睦、友爱互助的和谐乡村。

打好扶贫开发攻坚战。深入实施脱贫致富奔小康工程，抓好低收入人口增收、村集体增收、村服务中心达标“两增收一达标”，年内确保17万低收入人口和68个经济薄弱村实现脱贫。完善精准扶贫、精准脱贫责任制，抓好产业扶贫、智力扶贫、保障扶贫，建好阳光扶贫监管系统，健全扶贫对象动态调整机制，实现扶贫到村、到户。突出对丰县湖西革命老区和黄河故道沿线区域的项目扶持，推动惠民工程向集中连片贫困地区倾斜。加大财政投入，严格督促考核，充分发挥挂钩单位、帮扶工作队作用，提升结对帮扶和行业扶贫实效。调动贫困地区干部群众的积极性主动性，形成外部多元扶贫与内部自我脱贫的互动机制，决不让一个人在小康路上掉队！

（四）深入推进改革开放创新，形成强劲有力的发展新动能。习总书记强调，要坚持全面深化改革，坚持打开国门搞建设，加快建设创新型国家。我们要坚持以深化改革开放创新推动动能转换，为徐州发展开辟更大空间。

纵深推进体制机制改革。不断深化“放管服”改革，扩大相对集中行政许可权改革试点范围，分类实施综合行政执法体制改革；以“不见面”审批为抓手，完善政务服务“一张网”，巩固“3550”改革成果，推动政务服务更加优质、更加便捷。深化国企国资改革，全面推进市属国有企业重组整合，不断提升国有经济的引领和带动作用。深化财税金融体制改革，完善政府投融资机制，规范推行PPP模式，加强政府债务管理；稳妥推进市区农商行改革，积极申报国家级金融改革试点；加大不良贷款化解力度，提高防范化解金融风险能力。深化农业农村改革，继续抓好农村集体产权制度、承包地“三权分置”改革和农村金融创新，激发农村发展内生动力。深入推进“万村整治”工程，抓好“矿地融合”发展示范区建设，积极争取全国新一轮土地利用总体规划（2020-2035年）试点，争创全国国土资源节约集约模范市。实施新一轮“一地一策”牵引性改革，推动各板块大力进位争先。

全方位推进双向开放。积极主动融入“一带一路”建设，以打造开放型经济平台为抓手，加快创建沿东陇海线经济带双向开放改革试验区，争创国家服务外包示范城市、跨境电子商务综合试验区。启动“一带一路”中格共同市场合作区建设，抓好综合保税区、新沂保税物流中心（B型）建设。保障中欧班列稳定运行，高水平打造“无水港”，打通陆海内外联动新通道。继续实施观音机场改扩建，开辟新的国际航线，增加航班班次，申创国际快件中心，促进临空产业加快发展。完善国际贸易“单一窗口”功能，提升通关一体化水平，加快“外贸港”建设。积极推进国家生态原产地产品保护工作，提高出口产品的国际知名度和竞争力。坚持引进来和走出去并重，鼓励重点企业积极参与全球化进程，努力把徐工巴西制造基地打造成国家级对外投资合作典范项目。强化招商引资生命线地位，坚持领导带头招商，打造专业化招商队伍，办好第二十一届投资洽谈会、北京投资推介会、上海合作恳谈会、第二届国际服务外包大会等重点招商活动，确保招商引资实现更大突破。

高水平推进科技创新。加快完善区域性产业科技创新中心框架体系，努力建设国家知识产权示范市和国家创新型城市。抓好一流创新载体打造，江苏淮海科技城以资源整合为重点，加快“创客街”、2.5产业园等项目建设，推动科技企业、众创空间、孵化器、加速器加快集聚；徐州科技创新谷以功能完善为重点，实施主体区智慧小镇、国际学术交流中心等项目，吸引更多国内外一流研究院所落户；潘安湖科教创新区以基础建设为重点，加快推进科文学院、徐州幼专新校区建设，打造集人文特色、科技特色和生态特色于一体的城市新片区；产业技术研究院以成果转化为重点，深化与著名高校、大院大所对接合作，促进关键性、原创性和前瞻性技术成果加速实现市场化、产业化。抓好一流创新主体培育，发挥企业创新主导作用，推动骨干企业普遍建立研发机构，壮大高新技术企业集群；完善创新创业孵化体系和服务体系，支持中小微创新企业加快发展壮大。抓好一流创新人才引进培育，设立“彭城英才”投资基金，实施顶尖人才引进、领军人才集聚、双创人才倍增等计划，加大高层次人才资助、补贴、奖励力度，大力吸引诺贝尔奖、两院院士、国家千人计划等人才来徐发展，培育建设结构合理、作用突出的人才队伍，继续举办“赢在徐州”大赛，打造淮海经济区人才高地。

（五）提升生态建设水平，打造靓丽宜居的美好家园。牢记习总书记“打造绿水青山，并把绿水青山变成金山银山”的谆谆嘱托，以对人民群众高度负责的态度，全力推进污染防治和生态建设，切实转变发展方式，坚决不要带污染的GDP，闯出一条绿色生态发展新路子，把徐州建成生活富庶、身心愉悦、蓝天常在、绿水长流的美好家园！

着力解决突出环境问题。深入抓好中央和省环保督察组反馈意见整改落实，强力推进“263”专项行动。实施大气污染严管严控，强化工业、交通、建筑等多领域协同共治，有效降低PM2.5浓度，不断增加空气优良天数。持之以恒抓好水环境治理，深入落实“河长制”和“断面长制”，开展生态河湖专项整治，启动奎河综合治理工程，年内完成市区非建成区范围内19条黑臭河道整治，推进县（市）黑臭水体治理，实施城区污水管网及雨污分流工程，保持断面水质稳定达标。平稳有序推进减煤减化，持续开展化工企业“四个一批”专项行动，严格限制高能耗高污染项目落地，从源头上杜绝新增污染。加快关停“小散乱污”企业，推进钢铁、化工、水泥等行业企业重组转型。加大环境执法力度，严厉打击偷排漏排、设施不正常运行、非法跨界倾倒污染物等环境违法行为。

抓好生态保护和生态修复。积极融入江淮生态大走廊建设，筑牢全域生态安全屏障。抓好生态红线及流域综合监测系统建设，做好第二次全国污染源普查。统筹推进山水林田湖生态保护和修复工程，加快推进山体修复和塌陷地治理，支持沛县创建国家重点采煤沉陷区综合治理试点。开展全市农用地土壤污染状况详查，有序推进市区污染地块治理修复。实施植树造林工程，年内新增成片造林4万亩，全市林木覆盖率稳定在30%以上，让城市生态底色更加鲜明亮丽。

推进绿色低碳循环发展。做好能源消耗、温室气体排放总量和强度“双控”，在新能源发电、资源综合利用等领域实施一批能源结构调整项目，加快推进铜山燃气热电联产及南区热电、新汇热电煤改气项目，完成省下达的碳排放强度下降任务。推动前端清洁化生产和后端废弃物资源化利用，支持园区、企业实施绿色化、循环化改造，打造一批绿色产品、绿色工厂、绿色园区。加快循环经济产业园发展，抓好第二垃圾焚烧厂（一期）建设，推进餐厨废弃物统一收运处理，提高城乡生活垃圾无害化处理率。继续抓好国家节能减排示范市和循环经济示范市建设。

（六）着力保障和改善民生，一定让徐州人民生活更加幸福更加美好。习总书记在参观淮海战役纪念馆时指出，人民选择了共产党，没有共产党就没有新中国，今天我们共产党用什么来回报人民，那就是让人民过上幸福美好的生活。我们要始终把人民利益摆在至高无上的地位，多谋民生之利，多解民生之忧，加快构建社会建设“十二大体系”，不断提高公共服务水平，最大程度满足人民群众期盼。

加快完善公共服务体系。坚持优先发展教育事业，强化师资队伍建设，提升各级各类教育水平；加大中小学校、幼儿园建设力度，抓好幼儿看护点整改提升，促进基础教育优质均衡发展；办好职业教育，深化产教融合、校企合作；支持在徐高校建设一流大学和一流学科；健全社区教育网络，提升社会培训水平，完善全民学习终身教育体系。加快“健康徐州”建设，深化综合医改，推动医疗资源向基层下沉；推广家庭医生签约服务，加强计划生育服务管理，抓好群众性应急培训，守护群众身边的健康；培养引进高层次医疗人才，争创一批国家级临床重点专科，加快推进重点医院迁建和分院建设，提高医院装备、技术、医疗水平，建设更高水平医疗服务中心。加快老龄事业和产业发展，抓好全国居家和社区养老服务改革试点，建成一批老年日间照料中心、社区居家养老服务中心、老年助餐项目，办好老年大学。完善公共体育设施，倡导全民健身，促进群众体育和竞技体育融合发展，办好第二届中国徐州国际马拉松赛等体育赛事。繁荣文化事业和文化产业，深入实施文化惠民工程，加快完善公共文化服务设施，推出“汉乐华章”等一批文化精品力作，办好文博会，广泛开展全民阅读活动，打造“书香徐州”。

促进扩大就业和增加收入。实施更加积极的就业政策，发挥民营经济促进就业的主力军作用，完善覆盖城乡的公共就业服务体系，着力优化就业结构、提高就业质量。帮助重点群体就业，为高校毕业生提供多样化就业服务，统筹做好特困职工、返乡农民、下岗工人、退役军人等群体就业帮扶。鼓励以创业带动就业，开展多层次多元化创业培训，加大创业担保贷款支持力度，降低创业门槛和创业成本。深入实施“八项富民工程”，健全科学的工资水平决定机制、正常增长机制和支付保障机制，拓宽居民经营性、财产性收入渠道，实现居民收入增长与经济增长同步，让徐州人民的“钱包”尽快鼓起来。

努力提高社会保障水平。深入推行全民参保计划，完善多层次社会保障体系；推进城乡居民基本医疗保险全面并轨运行，实现市区基本照护保险参保全覆盖；将因病致贫对象及时纳入医疗救助，对灵活就业的就业困难人员发放社会保险补贴。完善社会救助、社会福利、慈善事业、优抚安置等制度，健全城乡居民低保标准、农村“五保”、城市“三无老人”和孤儿供养标准自然增长机制，加强困境儿童、留守儿童关爱保护。做好残疾人康复、托养、就业服务，将残疾人康复中心打造成行业示范机构。推动住房公积金归集扩面，建成经济适用房12万㎡、公租房9万㎡，健全住房保障体系。

继续加强和创新社会治理。深化平安法治徐州建设，落实社会治安综合治理领导责任制，打造共建共治共享的社会治理格局。推进“四项督查”常态化，深入开展“百千万”大走访，加强和改进信访工作，完善群众诉求表达和社会矛盾排查机制，从源头上防范化解各类风险隐患。加快建成立体化、信息化、智能化社会治安防控体系，依法打击和惩治黄赌毒黑拐骗等违法犯罪活动，努力打造淮海经济区最安全城市。加强社区综合服务中心建设，推行“全科社工”服务模式，推动社会治理重心下移、力量下沉。弘扬社会主义核心价值观，加强思想道德建设，完善社会信用体系，不断提升市民文明素质。牢固树立“生命至上、安全第一”的理念，完善安全生产网格化监管责任体系，提升应急处置能力，坚决遏制重特大事故发生。建设电梯安全公共服务平台。强化食品药品安全监管，打造区域放心消费环境。深化双拥共建，开展纪念淮海战役70周年系列活动，促进军民融合深度发展。

各位代表，解决民生问题是最大的政治，办好民生实事是政府最大的政绩。今年我们在广泛听取群众意愿的基础上，安排了11大工程75件实事，总投资540.8亿元。我们将全力以赴，确保办成办好，坚决兑现对全市人民的庄严承诺！

四、建设人民更加满意、更加信赖的人民政府

我们的政府是人民的政府。人民政府只有为人民，才能得到人民群众的拥护和支持。我们将始终坚持以人民为中心的发展思想，始终坚持全心全意为人民服务的宗旨，努力构建充满生机活力、更加廉洁高效的政务生态系统，让人民群众更放心、更满意、更信赖！

树立担当干事清廉的政府形象。弘扬敢于担当精神，营造想干事、能干事、干成事的浓厚氛围，不断提振工作精气神。完善政府工作推进、协调、督查体系，强化“钉钉子”精神，弘扬“马上就办”作风，在抓落实中推动各项工作见成效。健全鼓励激励机制和督查问责机制，为担当干事者撑腰、勇于创新者鼓劲，力戒推诿扯皮、敷衍了事、不作为乱作为，以奖惩分明促进勤政有为。深入一线问政于民、问需于民、问计于民，积极主动做好服务，以公务人员的辛苦指数赢得人民群众的满意指数、幸福指数。坚持无禁区、全覆盖、零容忍，坚持重遏制、强高压、长震慑，大力整治腐败问题，促进干部清正、政府清廉、政治清明。

打造优质高效的政务环境。深化简政放权、放管结合，以更彻底的放权、更严格的监管、更精准的服务，推动职能转变、释放市场活力。整合优化审批流程全链条，推行“不见面审批”，实行“多图联审”“多评合一”，最大程度减少审批时限。健全预审查、帮办代办等服务机制，清理规范涉审中介、强制性检验检测，清理减少审批费用，让企业和群众少跑腿、好办事。提高12345政府热线服务质效，完善市县一体化的公共资源交易市场，促进公共服务平台提质扩容。

不断提高依法行政水平。坚持领导干部带头学法尊法守法用法，运用法治思维和法治方式推动工作，把政府活动全面纳入法治轨道。加强合法性审查和风险评估，健全政府科学决策、民主决策、依法决策机制。认真听取人大代表、政协委员意见建议，充分听取民主党派、工商联、无党派人士和各人民团体的意见，提高人大代表议案建议和政协提案的办理质量。依法接受市人大及其常委会的监督，主动接受市政协的民主监督，重视司法、舆论、社会监督，完善审计监督机制。全面推行政务公开，完善新闻发布制度，让行政权力在阳光下运行。

持之以恒正风肃纪。凡是群众反映强烈的问题都要严肃对待，凡是损害群众利益的行为都要坚决纠正。开展“不忘初心、牢记使命”主题教育，推进“两学一做”学习教育常态化制度化，用党的创新理论武装头脑、指导实践。严守政治纪律和政治规矩，认真履行“一岗双责”，更高标准抓好党风廉政建设，把全面从严治党要求落实到政府工作的各方面。深入落实中央八项规定实施细则，厉行勤俭节约，严控“三公”经费支出，驰而不息纠正“四风”，力戒形式主义、官僚主义新“十种表现”。加强纪律教育和纪律执行，让党员、干部知敬畏、存戒惧、守底线，习惯在受监督和约束的环境中工作生活。

各位代表，徐州正处在建设淮海经济区中心城市的关键时期，我们肩负着新时代赋予的光荣使命。让我们更加紧密地团结在以习近平同志为核心的党中央周围，在中共徐州市委的坚强领导下，团结依靠全市人民，务实创新，埋头苦干，为打造“强富美高”新徐州、建设淮海经济区中心城市，决胜全面建成小康社会、开启现代化新征程而努力奋斗！
